# Hand Preference and Performance in Basketball Tasks

**DOI:** 10.3390/ijerph16224336

**Published:** 2019-11-07

**Authors:** Emanuela Gualdi-Russo, Natascia Rinaldo, Alba Pasini, Luciana Zaccagni

**Affiliations:** 1 Department of Biomedical and Specialty Surgical Sciences, Faculty of Medicine, Pharmacy and Prevention, University of Ferrara, 44121 Ferrara, Italy; emanuela.gualdi@unife.it (E.G.-R.); natascia.rinaldo@unife.it (N.R.); 2 Centre for Exercise Science and Sport, University of Ferrara, 44123 Ferrara, Italy

**Keywords:** hand preference, team sports, game role, game level

## Abstract

The aims of this study were to develop and validate an instrument to quantitatively assess the handedness of basketballers in basketball tasks (Basketball Handedness Inventory, BaHI) and to compare it with their handedness in daily activities by the Edinburgh Handedness Inventory (EHI). The participants were 111 basketballers and 40 controls. All subjects completed the EHI and only basketballers filled in the BaHI. To validate the BaHI, a voluntary subsample of basketballers repeated the BaHI. Exploratory and confirmatory factor analyses supported a two-factor model. Our results show that: (i) Handedness score (R) in daily actions did not differ between basketball players (R by EHI = 69.3 ± 44.6) and the control group (R by EHI = 64.5 ± 58.6); (ii) basketballers more frequently favored performing certain sport tasks with the left hand or mixed hands (as highlighted by R by BaHI = 50.1 ± 47.1), although their choice was primarily the right hand in everyday gestures; and (iii) this preference was especially true for athletes at the highest levels of performance (R by BaHI of A1 league = 38.6 ± 58.3) and for those playing in selected roles (point guard’s R = 29.4 ± 67.4). Our findings suggest that professional training induces handedness changes in basketball tasks. The BaHI provides a valid and reliable measure of the skilled hand in basketball. This will allow coaches to assess mastery of the ball according to the hand used by the athlete in the different tasks and roles.

## 1. Introduction

Studies on cerebral dominance have found that the left hemisphere is not only dominant for speech but also for the selection of actions and the translation of these responses into action [1]. Almost 50 years ago, Oldfield [2] proposed the Edinburgh Handedness Inventory (EHI) as a method to assess handedness in everyday activity on a quantitative scale, which is still the most widely used method in assessing laterality. According to a recent review [3], 899 published articles used this method, albeit by modified versions of the EHI. A trichotomous assessment of lateral preferences was recently suggested [4,5] since considering only the left or right preference excludes mixed handers from the analysis with a significant loss of information.

Although a large majority of humans are right-handed (85% to 93% according to Raymond and Pontier [6]), left-handedness is believed to lead to some tactical advantages in those sports in which there is an opponent, such as, for example, boxing [7,8], wrestling [9], badminton [10], cricket [11], tennis [12,13], baseball [14], and volleyball [15], as well as for team sports with direct contact among players, such as basketball [16,17], football [17,18,19], rugby [17], or water polo [20]. Conversely, in hockey and polo, the rules of the sport itself explicitly disadvantage left-handers; in polo, the mallet has to be held in the right hand on the right side of the horse, while hockey sticks come only in a right-handed form and must be held right-handed [18,21]. Some evidence of left-handers’ advantage resulted especially when they played against teams that might have less experience with left-handers [22]. One explanation for this phenomenon is the negative frequency-dependent selection hypothesis proposed by Faurie and Raymond [23]. They supposed that the lack of familiarity with the playing techniques and tactical strategies of left-handed competitors may disadvantage a player acting in the same way as being faced with a right-hander; moreover, the outcome of left-handed players’ actions can be predicted with a significantly lower accuracy compared to right-handers’ actions [15,24,25].

Other explanations, such as the fighting hypothesis [9,26], are considered to be not supported by consistent results [27]. Conversely, no prevalence of left-handedness was observed in the case of sports without interacting opponents, such as golf or darts [6,28].

Only a few studies have been performed to quantify hand preference in basketball players and how much experience is needed to play with the non-preferred hand [29,30,31]. Using archival data, the prevalence of left-handers was 5% in professional players (period 1946–2009) vs. 11% in the general population [29]. Stockel and Weigelt [30] found in their analysis a tendency of professional players towards a more frequent use of the non-dominant hand during the game and a reduction to about 49% in the use of the dominant hand in professional players vs. 59% in amateurs. Considering the hand preference in everyday life handedness by professional players, there was a reduced one-hand bias due to basketball training of both the dominant and non-dominant hand or to an advanced selection process with less lateralized individuals, since a proficiency with both hands can be advantageous in this sport [31,32]. None of these researches proposed a specific questionnaire leading to a quantitative scale of scores comparable to EHI while evaluating, at the same time, the laterality in the typical tasks of basketball.

Hypothesizing that a strong lateralization may be a disadvantage in basketball, as previously alleged [29,30,31], we decided to evaluate it by a new inventory based on as many items as the expected skills, providing a total score for basketball handedness comparable to the one achieved in daily activities (EHI). Once the new inventory was validated, we aimed to verify: (i) Hand preference in everyday activities of basketball players compared to controls; (ii) the relation between everyday laterality and basketball laterality; and (iii) the influence of the competitive level, role, and training on basketball laterality.

## 2. Materials and Methods

### 2.1. Participants

Athletes were recruited from Italian basketball leagues. A total of 111 male basketball players (age: 23.7 ± 4.8 years) were enrolled in this study. They have been practicing this sport on average for 12.1 ± 5.1 years within an organized team with an average training of 11.7 ± 6.4 h/week. These players participated in the 2016–17 season of the Italian A1 (N = 23), A2 (N = 21), and B (N = 30) leagues while 37 of them participated in an amateur basketball league. Taking into account the different tasks performed by the players in relation to their role, the players were divided into five groups according to the game role: Twenty-four point guards, 30 shooting guards, 22 small forwards, 21 power forwards, and 14 centers. We approached amateur players directly at sports associations in the Ferrara and Rovigo area (North-Eastern Italy) while professional players were indirectly contacted via coaches and secretariats of Italian basketball teams and individually compiled inventories, after the distribution, on a voluntary basis.

In addition, we recruited 40 control subjects (male students of the University of Ferrara who agreed to participate in this study) via a convenience-based sampling approach. Following a general announcement, students interested in participating received full information on the research. The unique inclusion criterion was to not be engaged in any sport activity, in order to avoid any interference with laterality caused by other specific sport activities. The control group was 25.1 ± 2.3 years old.

Before participating in this study, each participant filled out a brief questionnaire about his sporting background to be used in the selection of non-athletic controls in addition to the main personal information. Furthermore, data on sports practice were collected for basketball players, indicating the game role, current competitive level, years of coached practice, and hours/week training.

The study was conducted in accordance with the Declaration of Helsinki, and the protocol was approved by the Ethics Committee of Ferrara University, Italy. All participants, fully informed about the study aims and procedures, provided written consent before taking part in the study. Apart from signing the informed consent, there were no further exclusion criteria.

### 2.2. Procedures

In order to determine the dominant hand in everyday life, the EHI [2] was used both for athletes and controls compiling the relevant items.

**Figure 1:**
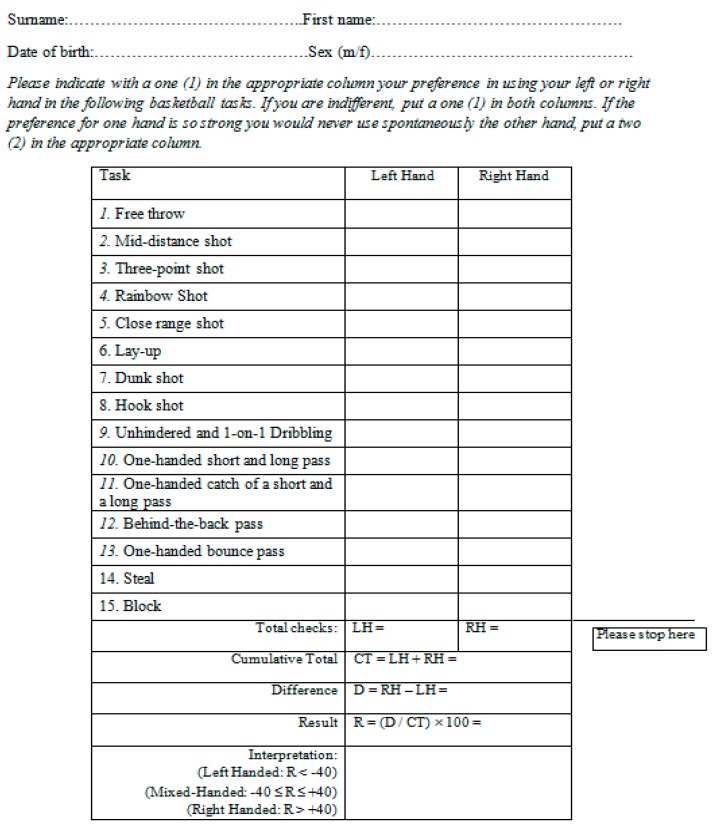
Basketball Handedness Inventory (BaHI) with instructions.

To determine the hand preference in basketball tasks, a new inventory with basketball-specific items on hand preference was used (BaHI: Basketball Handedness Inventory). This 15-item inventory was developed for this study on the basis of seven tasks suggested in a previous questionnaire on laterality in basketballers [31], and a further eight tasks reported after consultation with a panel of three experts in basketball and sport sciences (Figure 1).

These items were entered into a format and scoring method similar to EHI to obtain a quantitative assessment and interpretation of laterality in basketball. The procedure was as follows. The response sheet presents two columns—‘left hand’ and ‘right hand’—following each item. Similar to the EHI procedures, the participant indicated the number 1 (weak preference to one hand) or 2 (strong preference to one hand) in the appropriate column box to indicate their preference for the right or left hand for a certain task. If there was no particular hand preference for an item, the participant ticked 1 in both columns. At the end of the procedure, a score of laterality was computed as R = (difference between sides/cumulative total) × 100, ranging from −100 (strongly left) to +100 (strongly right). Scores between −40 and +40 were interpreted as the mixed handedness condition as suggested by Stockel and Vater [31]; and scores lower than −40 and greater than +40 were interpreted, respectively, as left-handed and right-handed.

Four weeks later after filling out of the new inventory, we asked all basketball players to again complete the BaHI to estimate the test–retest reliability. A subsample of 20 basketballers agreed to participate in the repetition.

### 2.3. Statistical Analysis

In order to assess BaHI validity, we first evaluated its internal consistency by Cronbach’s alpha coefficient. To determine test–retest reliability, we examined the intraclass correlation coefficient (ICC) between corresponding R scores of the initial test and retest. We applied a two-way model based on single measures, with the same raters for all subjects and consistency. An ICC value of more than 0.80 was considered to indicate a high test–retest reliability for a measure [33]. In addition, Bland Altman plots with 95% limits of agreement were made to observe the variance between repeated assessments [34]. Correlation analyses were conducted to examine the association between the items. We conducted an exploratory factor analysis (EFA) on a subsample of 20 basketballers who repeated the inventory. In this analysis, responses for each item of the BaHI were recorded in data set as 0 = indifferent hand preference, 1 = right-hand preference and 2 = left-hand preference. EFA, using the principal factor method and varimax rotation procedure, was conducted on the 15 items of BaHI to determine its factor structure. The Scree plot of eigenvalues was examined to determine the number of factors to retain in the model. Then, we used confirmatory factor analysis (CFA) in the group of 91 basketballers who did not repeat the inventory to confirm the factor structure generated by EFA. The fit of the model was examined in terms of chi-squared (CMIN), degree of freedom (df), comparative fit index (CFI), and root mean square residual (RMR).

In the sample analysis, all data are expressed as means ± SD and frequencies. According to Oldfield [2], the lateral preference in everyday life was determined by laterality index scores comparing groups (athletes and controls) by the Mann–Whitney U-test. Comparisons among game roles (point guard, shooting guard, power-forward, small forward, and center) and divisions (A1, A2, B, and amateur) were carried out by Kruskal–Wallis ANOVA. Comparisons between everyday life handedness and basketball handedness on the same basketball players were performed by the Wilcoxon signed-rank test. The frequency distribution of lateral preferences was compared between groups by the Chi-square test. The association between the basketball handedness score and other quantitative variables was investigated using the Spearman correlation coefficient. A *p*-value < 0.05 was considered to indicate statistical significance. The data analysis was performed using Statistica for Windows (version 11) (StatSoft Inc. Tulsa, OK, USA) and AMOS 26.0 (IBM, USA).

## 3. Results

### 3.1. Validation of BaHI

**Figure 2:**
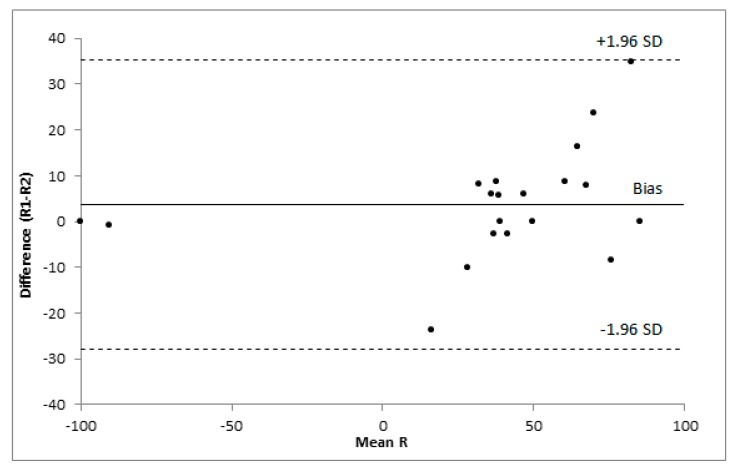
Bland–Altman plot: graphical display of the means against their respective paired differences, the Bland–Altman limits of agreement (dashed lines), and the bias, i.e., estimated mean difference (solid line).

The BaHI demonstrated a good internal consistency. Cronbach’s alpha coefficient was 0.892. The ICC for the BaHI was 0.92 (95% CI = 0.80 – 0.97). In the Bland–Altman analysis, the mean difference of R scores for test–retest measurements was 3.8 (SD 16.1; 95% limits of agreement = −27.8 to 35.4). All the differences between the two repetitions were between the limits of agreement (Figure 2).

In all cases but three (block vs. one-handed bounce pass; block vs. one-handed short and long pass; one-handed short and long pass vs. steal), the bivariate correlation between items was statistically significant (Table 1).

**Figure 3:**
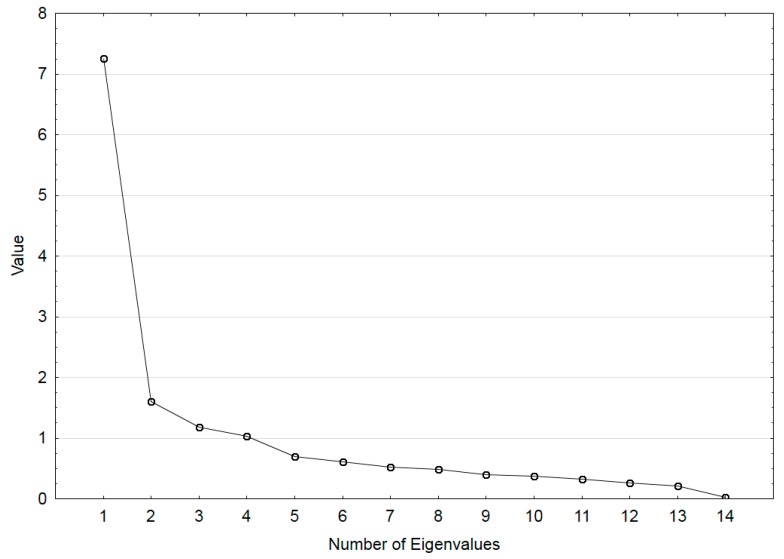
Scree plot for the BaHI items.

The Scree plot of eigenvalues (Figure 3) indicated a two-factor model, accounting for 59% of the total variance (Table 2). Factor 1 accounts for 48% of the variance and, based on the included skills with the greatest loadings (free throw, mid-distance shot, three-point shot, rainbow shot), it can be called a construct of “distance shots”. Four tasks (steal, block, one-handed catch of a short and a long pass, and behind-the-back pass) had the greatest loadings on factor 2, accounting for 10% of the variance. Based on the included tasks, this factor can be named a construct of “defensive recovery”.

The factor structure of the BaHI identified through EFA was subjected to a CFA in the group of those who did not repeat the inventory. The model derived from the EFA showed a good fit with the data (CMIN = 1.449; df = 19; CMIN/df = 0.076; CFI = 1.000; RMR = 0.021). According to conventional criteria, the chi-squared/df < 2, CFI > 0.9, and RMR < 0.05 indicate a good fit [35,36]. 

### 3.2. Assessment of Hand Preference

The descriptive data on everyday laterality preference for basketball players and controls show a higher rate of right-handers in both samples (basketballers: R 86.5%, L 4.5%, mixed 9%; controls: R 85%, L 10%, mixed 5%). Although a greater number of mixed handers was found among players compared to controls, no statistical difference was identified in everyday life handedness based on EHI or in R scores (basketballers: R = 69.3 ± 44.6 scores; controls: R = 64.5 ± 58.6 scores). 

**Figure 4:**
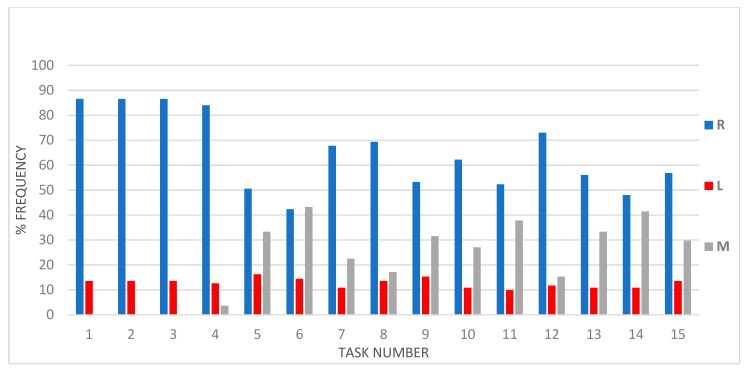
Frequency histogram showing per cent right-handed (R), left-handed (L), mixed handed (M) players in the 15 tasks of the BaHI inventory (on the x-axis).

Figure 4 shows the distribution of handedness for the 15-item inventory in basketball players. Right-handers are prevalent in all skills, except in layup. A high frequency of left-handers is observed also in the steal action. Mixed-handed are more frequent in close range shot action, followed by unhindered and one-on-one dribbling and block. No mixed-handed resulted in the first three items. In order to identify possible differences between every day and basketball handedness, we examined the distribution of overall hand preference according to BaHI among the 111 expert players classified as right-handed, left-handed, or mixed handed in daily activities (Table 3).

The distribution differed significantly, with a 20% decrease of right-handers from daily activities to basketball tasks. The average R score decreased from 69.3 ± 44.6 in everyday handedness to 50.1 ± 47.1 in basketball handedness (*p* < 0.0001). Although we found higher frequencies of left-handed and, especially, mixed handed and a lower frequency of right-handed A1 players than in other basketball professional players, and the R scores were, on average, lower in players of the A1 league than in other leagues, no significant differences were found (Table 4).

The frequencies of handedness differed significantly among game roles, with more left-handers and mixed handed point guards and small forwards. Consistently, we found R scores were significantly different among game positions (*p* = 0.0252), with the lowest values in point guards and small forwards and the greatest one in centers.

Correlation analyses were performed to determine the strength and direction of association between hand preference in basketball (BaHI R score) and years of basketball practice, amount of weekly training (average for the last year), and hand preference in everyday activities (EHI R score) in the whole sample of players or only in the subsample of professionals. We found no significant association between BaHI R score and years of practice in any case. BaHI R score was significantly and negatively associated with weekly training only in professional athletes (r = −0.316, *p* = 0.006). The last variable (EHI R score) was positively and significantly correlated to the BaHI R score in both cases (r = 0.411, *p* < 0.0001 in the whole sample; r = 0.541, *p* < 0.0001 in professional players).

## 4. Discussion

In this study, we developed a new instrument (BaHI) to assess the handedness of basketballers in basketball tasks by using an assessment system similar to the one generally used for everyday activities (EHI), thereby making a comparative survey of manual dominance both in everyday life and in sport possible. Through a conventional analytic approach, we demonstrated the validity and reliability of this new inventory on the handedness of basketball tasks, consisting of 15 items. The CFA confirmed the exploratory model in a larger sample of players.

Second, we analyzed handedness differences between basketball players and controls in everyday activities and, among athletes, we examined handedness differences according to the league and game role. The handedness of players in everyday life and basketball tasks was compared through the scores obtained in the two inventories. The main findings are the following: (i) Handedness in daily activities was similar in athletes and controls; (ii) basketball athletes performed certain sport tasks preferably with the left hand or mixed hands, and they were also right-handed in everyday life; and (iii) the handedness in basketball tasks correlated positively to the handedness in daily activities and negatively to sport practice (amount of weekly hours).

In contrast to the results of Stöckel and Vater [31] on professional basketball players compared to a general population [37], we did not find differences in everyday handedness between the self-reported data of players and controls. The examined basketball players reported a greater right-hand preference and a lower left-hand or equal-hand preference than the sample of Stöckel and Vater [31]. In addition to differences in size between the samples of the two studies, our sample included the three highest Italian leagues and an amateur sample, which was not present in the German study. Moreover, the Italian sample was one year younger, on average, compared to the other one, while the years of coached practice were similar. Given the importance of the role, it is also possible that a different composition of the two samples may have influenced the results. In this respect, by classifying our players into only three playing positions to compare their frequencies to those in the cited German study, there were 48.6% of guards vs. 76%, 38.7% of forwards vs. 34.3%, and 12.6% of centers vs. 20.7%. A guard frequency higher than 27% in the German sample compared to the one of our sample may, in our opinion, have influenced the results as the point guards show the highest frequency of left-handed and mixed handed in sports tasks. However, according to our study, this tendency was detected only in sport tasks, without any influence on everyday actions.

The proficiency of expert basketball players in passing and shooting skills with both the dominant and non-dominant hand [30] was confirmed by the significantly lower R scores achieved by the players in the sport tasks (BaHI R) compared with daily life actions (EHI R). Trained to also use the non-dominant hand in this sport, the different handedness detected in sport tasks is conditioned by the need to use the most proficient hand in different situations in the game, especially when there is an interaction with other players or when the player is under pressure [31]. As highlighted by the first three tasks, the use of the dominant hand is confirmed in pressure-free situations and in the absence of direct opposition. Although an association between hand preference in everyday life and in basketball was found in our study, an adaptation in hand preference to basketball demands is consistently different from other studies [30]. A proficiency of both hands in basketball is very advantageous, as the low R scores of the players of the A1 league seem to indicate.

We also investigated factors that are possibly associated with handedness in the basketball practice, but we did not find any significant association of handedness with performance (assessed by the belonging league) and years of practice. Conversely, the handedness in everyday life activities and weekly training (among professional players) was predictive of basketball handedness. According to literature [38,39,40], early training in sports leads to advantages and physiological adaptations. Although individuals with more coached practice should have had more opportunities for necessary physiological adaptation in relation to their playing position and thus be more skilled, based on our findings, the BaHI R decreased significantly as the amount of training increased, but not significantly as the years of practice increased. 

The current study presented a new method to quantitatively assess handedness in basketball tasks, ensuring the possibility of comparisons with EHI outcomes by R scores. The main limitations of this study are the following. Only one fifth of the participants agreed to repeat the test. Due to the cross-sectional study design, possible changes in handedness of the basketball player during the sport practice period could not be assessed. Furthermore, the results of this study cannot be extended to females, since female players were not included in this survey. Lastly, although we selected controls that did not play sports, we cannot exclude that other activities practiced by them may have influenced their laterality.

## 5. Conclusions

In this study, we proposed a new inventory, the BaHI, to provide a reliable measure of handedness in basketball skills for coaches to assess hand proficiency in basketball tasks. The findings obtained using the BaHI have led us to hypothesize that professional training could change hand preference in basketball tasks, especially in players at the highest level of performance and in some game roles.

The BaHI should be used to monitor players in relation to their playing role and to evaluate possible changes in movement skills in very young basketball players (children, adolescents).

To confirm our findings and further elucidate the interactions between handedness and athletic skills, further research is needed using the novel and readily applicable inventory.

## Figures and Tables

**Table 1:** Bivariate correlations between the items of the BaHI.

No Item	1	2	3	4	5	6	7	8	9	10	11	12	13	14	15
**1**	-														
**2**	1.00	-													
**3**	0.96	0.96	-												
**4**	0.71	0.71	0.67	-											
**5**	0.41	0.41	0.46	0.56	-										
**6**	0.54	0.54	0.51	0.62	0.67	-									
**7**	0.45	0.45	0.51	0.45	0.43	0.52	-								
**8**	0.55	0.55	0.52	0.64	0.50	0.59	0.48	-							
**9**	0.41	0.41	0.46	0.40	0.42	0.39	0.36	0.35	-						
**10**	0.46	0.46	0.42	0.41	0.34	0.40	0.37	0.31	0.53	-					
**11**	0.47	0.47	0.51	0.39	0.33	0.44	0.56	0.43	0.49	0.52	-				
**12**	0.54	0.54	0.50	0.41	0.43	0.46	0.45	0.53	0.48	0.54	0.60	-			
**13**	0.33	0.33	0.28	0.31	0.33	0.48	0.35	0.34	0.36	0.50	0.45	0.59	-		
**14**	0.23	0.23	0.26	0.21	0.23	0.28	0.34	0.22	0.30	0.15^ns^	0.48	0.39	0.27	-	
**15**	0.23	0.23	0.27	0.20	0.21	0.28	0.39	0.26	0.23	0.14^ns^	0.37	0.39	0.14^ns^	0.54	-

1. Free throw; 2. Mid-distance shot; 3. Three-point shot; 4. “Rainbow” Shot; 5. Under The Basket Shot; 6. Layup; 7. Dunk; 8. Hook; 9. Unhindered and one-on-one Dribbling; 10. One-handed short and long pass; 11. One-handed catch of a short and a long pass; 12. Behind-The-Back Pass; 13. One-Handed Bounce Pass; 14. Steal; 15. Block. ^ns^ = not significant.

**Table 2:** Factor loadings for each item of the BaHI in basketball players.

Item	Factor 1	Factor 2
1. Free throw	0.9298	0.1359
2. Mid-distance shot	0.9298	0.1359
3. Three-point shot	0.8954	0.1772
4. Rainbow Shot	0.8225	0.1702
5. Close range shot	0.5574	0.3432
6. Lay-up	0.6313	0.4125
7. Dunk shot	0.4517	0.5438
8. Hook shot	0.6501	0.3194
9. Unhindered and 1-on-1 Dribbling	0.4220	0.4934
10. One-handed short and long pass	0.4729	0.4303
11. One-handed catch of a short and a long pass	0.3851	0.6974
12. Behind-the-back pass	0.4597	0.6513
13. One-handed bounce pass	0.3054	0.5622
14. Steal	0.0228	0.7538
15. Block	0.0497	0.6757
*eigenvalue*	*7.26*	*1.60*
*% variance*	*48.41*	*10.68*

**Table 3:** Distribution of basketball players (N = 111), classified by EHI, within each category of hand preference according to BaHI (percentages in parenthesis).

Handedness	by BaHI		
by EHI	Right	Left	Mixed Handed
Right	77 (69.4%)	3 (2.7%)	16 (14.4%)
Left	1 (.9%)	5 (4.5%)	0 (0%)
Mixed handed	2 (1.8%)	4 (3.6%)	3 (2.7%)

**Table 4:** Absolute and percent (in parenthesis) frequencies within each category of hand preference (BaHI inventory) and R scores among basketball divisions (above) and roles (below).

**Handedness**	**A1**	**A2**		**B**	**Amateur**
Right-handed	16 (69.6%)	16 (76.2%)		24 (80.0%)	25 (67.6%)
Left-handed	3 (13.0%)	2 (9.5%)		3 (10.0%)	3 (8.1%)
Mixed handed	4 (17.4%)	3 (14.3%)		3 (10.0%)	9 (24.3%)
*R score mean (SD)*	*38.6 (58.3)*	*51.6 (51.6)*		*58.4 (58.1)*	*49.6 (57.5)*
**Handedness**	**point guard**	**shooting guard**	**small forward**	**power forward**	**center**
Right-handed	14 (58.3%)	26 (86.7%)	12 (54.6%)	16 (76.2%)	13 (92.9%)
Left-handed	5 (20.8%)	0 (0%)	3 (13.6%)	3 (14.3%)	0 (0%)
Mixed handed	5 (20.8%)	4 (13.3%)	7 (31.8%)	2 (9.5%)	1 (7.1%)
*R score mean (SD)*	*29.4 (67.4)*	*67.5 (26.2)*	*33.7 (57.0)*	*50.5 (63.9)*	*73.1 (20.3)*
